# A systematic review of school-based student peer-led oral health interventions to promote the oral health of school children

**DOI:** 10.1186/s12903-023-03482-1

**Published:** 2023-10-10

**Authors:** Yasmen E. Elsadek, Sakina Edwebi, Abigail Turner, Karen Vinall-Collier, Julia Csikar, Sue Pavitt

**Affiliations:** School of Dentistry, Faculty of Medicine and Health, University of Leeds, Leeds, UK

**Keywords:** Health promotion, Peer-led, School-based, Oral health

## Abstract

**Background:**

Poor oral health in children highlights the need for prevention and effective interventions. During late childhood and adolescence, peer relationships can play a vital role in adopting and maintaining positive health behaviours.

**Aim:**

To identify the oral health outcomes of school-based student peer-led delivery of oral health interventions.

**Methods:**

A search strategy was developed, piloted, and run in four electronic databases: Medline via Ovid, Web of Science, CINAHL via EBSCO, and CENTRAL (Cochrane Central Register of Controlled Trials) using key concepts of peer, oral health and adolescent in the school context. Methodological quality was assessed using QuaDs quality assessment tool. All articles were independently screened by two researchers and data was analysed using narrative data synthesis. The PRISMA checklist complemented by aspects of the Synthesis Without Meta-analysis (SWiM) was used to report this systematic review.

**Results:**

There were 7572 identified, 24 studies progressed to full-text review, ten studies met the eligibility criteria and were included in the review. Only six studies based their interventions on psychological & behavioural theory. Intervention delivered by peers showed improvements in both clinical and self-reported outcomes when compared to other delivery methods (e.g., professionals). Quality of included studies was reported according to QuaDs guidance.

**Conclusion:**

Peer-led interventions were more effective in improving oral health status and behaviours when compared to other modes of delivery. Future research should assess if a bi-directional impact of peer-led interventions can be seen. Specifically, if there is added value for school-based student peer-leader's including their own oral health knowledge, skills, attitude and preventative behaviours.

**Supplementary Information:**

The online version contains supplementary material available at 10.1186/s12903-023-03482-1.

## Introduction

The burden of tooth decay amongst children is a significant public health challenge and a priority, with more than 530 million children suffering from tooth decay of primary teeth [1]. Over the past decade, advances in prevention strategies have led to a steady decline in the number of children with tooth decay [2]. However, oral diseases are disproportionally higher in those from socially disadvantaged backgrounds and increasingly concentrated in high-risk groups within socioeconomically deprived areas [3]. Tooth decay in children presents a considerable health, economic and social burden, affecting school attendance with a minimum of 60,000 days missed from school per year in the UK due to dental pain [4]. The correlation between inadequate oral hygiene during childhood and poor oral health in adulthood is widely recognized [2]. Poor oral health during childhood can have long-term consequences, research has shown that adults who experienced tooth decay during their childhood are more likely to continue to experience poor oral health later in life [2]. According to UK national epidemiological survey, nearly half of the young children who begin secondary school have decayed teeth decayed [5]. This highlights a significant health concern that needs to be addressed urgently [4].

Implementation of oral health promotion and improvement programmes is common within the school environment [6]. Schools have long been proposed as an opportune entry point for general and oral health improvement interventions, the World Health Organisation (WHO) emphasised the importance of promoting health in school settings since the 1980s [6]. Healthy behaviours, attitudes and skills are established at a young age, and therefore schools are recognised as an ideal environment to influence a child’s development and wellbeing. The success and sustainability of school-based interventions is often affected by multiple factors, such as the capacity of school to enable interventions to be delivered, staff motivation and the existing educational commitments which the school must prioritise, time allocated for delivery, funding and material resources [7]. Conflicting priorities in the school, lack of time, or an over-reliance on a single co-ordinator has been shown to influence the success of the programme [8].

Student-led interventions for health promotion are increasingly common (often referred to as ‘peer-led’). These strategies are utilised to target a range of health outcomes including weight loss, smoking cessation, breast cancer, mental health, diabetes and addiction recovery [9]. The predominant rationale for the use of peer-led interventions stems from the social influence theoretical model. The premise proposed [10] is that “friends seek advice from friends and are also influenced by the expectations, attitudes and behaviours of the groups to which they belong” (9 p.187). These interventions rely on the credibility and shared cultural background of the ‘leader’ or ‘peer’. These leaders may act as a positive role model which can aid the underpinning of behavioural messages [11]. A person, particularly children, can increase their self-efficacy by learning new knowledge and skills for handling situations through the observation of others. Ultimately, student led interventions are seen as a stronger influence on behaviours than those that are delivered by adults such as teachers or experts [12].

Previous reviews showed that school-based peer-led interventions in a variety of different contexts and addressing a range of health issues can achieve a change, in both health status, and behaviours [11–15]. However, research regarding oral health improvement via school-based peer-led interventions is still very much in its infancy. Hence, there is a need to focus on which peer interventions are the most effective for health improvement and particularly, oral health improvement [16]. The review aims to identify the oral health outcomes of school-based student peer-led delivery of oral health interventions.

## Methods

An initial search of PROSPERO, the JBI database of systematic reviews and Cochrane Database of Systematic reviews was performed to ensure there was no ongoing systematic reviews on the same topic. Subsequently, the systematic review protocol was registered with PROSPERO in October 2021 (registration number CRD42021283542) [17] and the 27-item PRISMA checklist [18] was used to report this systematic review.

### Search strategy

Key search terms were developed within the research team and through consultation with librarians and a full search strategy was created. The key search terms were: Oral health AND intervention AND peer-led AND school. (See additional file 1 for detailed search strategy).

The review followed a comprehensive systematic search of published literature from several databases: Medline via Ovid, Web of Science, CINAHL via EBSCO, and CENTRAL (Cochrane Central Register of Controlled Trials). The search was first conducted in October 2021, re-run in in August 2022 and again in September 2023 with no limits applied on publication date or country. Handsearching of reference lists and citation tracking were also carried out. Additionally, experts were contacted for identification of key studies in field that could have been missed after completing the search.

### Eligibility criteria

Interventional studies of peer-led oral health promotion interventions in 6–19-year-olds in school settings which reported oral health outcomes were included in this review. Interventions had to be based in an educational setting and delivered by students. The study design had to include a control or comparison group (i.e., dental professional-led, teacher-led, or self-learning), as well as pre- and post-intervention assessments to identify the effectiveness of the intervention. Not only educational interventions which provided information on improving oral health, diet and preventing oral disease were included but also those supplemented by behaviour change techniques and innovative tools for implementation and dissemination. However, interventions targeting specific groups (e.g., preschool children, specific chronic illnesses, or disabilities) were excluded. Table 1 provides details to justify the eligibility criteria.

**Table1:** Eligibility criteria with justification

	**Criteria**	**Justification**
**Inclusion**	Primary and secondary schoolchildren (aged > 6–19)	Focuses on school-aged children
	Interventions that are delivered by students (peers)	This review investigates the potential of peer (student-led) interventions
	Studies that compare peer-led interventions to another mode of delivery	to identify interventions that bring about a change in outcomes
	School-based interventions in both public & private schools	This review seeks to capture experiences in all school environments
	Studies in which oral health is their focus or part of their focus	To ensure an understanding of the impact of peer (student) led interventions on oral health
**Exclusion**	Pre-schoolchildren (< age 6) and children with disabilities	The review aims to explore interventions with implications for school-based populations. Targeted interventions for different conditions and younger age groups may require different strategies
	Interventions not delivered by students (peers)	This review investigates the potential of peer (student-led) interventions
	Any study where a comparator is not identified	To facilitate comparison between different modes of delivery
	Study conducted in a setting other than schools (e.g., community-based, healthcare setting)	This review seeks to capture experiences in school environments
	Studies which do not have oral health as their focus or part of their focus	To ensure an understanding of the impact of peer (student) led interventions on oral health

### Study selection

The search results were exported to EndNote where title and abstract screening was conducted, and duplicates were removed. Three authors (YE, SE, and AT) screened titles and then abstracts according to the inclusion criteria. Full text of the articles which met the inclusion criteria were then screened by YE and AT for inclusion. Any disagreements were resolved through discussion with the wider research team to reach a consensus. Reference lists of each article were hand-searched for any relevant studies that met the inclusion criteria.

### Data extraction

Data were extracted from included studies using a form developed by the authors to capture key information on populations, intervention strategies and results. Microsoft excel data extraction form was developed and pilot tested by two independent reviewers (YE and SE) using two samples of the included studies. Minor changes to the data extraction form were discussed by the research team to reach consensus and capture key information required to address the research question.

Data for all included studies were extracted independently by two reviewers (YE and AT). The data fields included were author (year), country, study design, participants information, sample size, aims, outcome measures, selection of peers, intervention type and components, intensity of intervention, duration/ follow up, intervention facilitator, key findings, theoretical model. Any discrepancies in data collected were resolved by discussion. When consensus could not be reached between reviewers, arbitration with the wider research team was undertaken. A summary of the extracted information is shown in Table 2.

**Table 2:** Characteristics of included studies

Study ID (country)	Sample size	Intervention	Comparator	Selection of peers	Follow up	Theoretical model
Laiho et al. (1993) (Finland) [19]	357	45 min Education & free toothbrushes delivery & xylitol chewing gum	Dentist-led & Self-teaching	Selected by school	2 weeks & 2 months	No
Haleem et al. (2012) (Pakistan) [20]	1517	1 h education and daily toothbrushing	Dentist-led, Teacher-led & Self learning	Nominated by teacher in charge	6,12,18 &24 months	Social cognitive theory
Haleem et al. (2015) (Pakistan) [21]	935	1 h education with daily toothbrushing vs reinforcement on monthly basis	Teacher-led & dentist-led	Nominated by teacher	6 months after 1 session. 6, 12 months post-reinforcement	Social learning theory
Debby et al. (2016) (Indonesia) [22]	70	10 educational sessions across 4 weeks	Dentist-led	Selected by teacher	4 weeks	No
Vangipuram (2016) (India) [23]	450	20 min educational session	Dentist-led	Not mentioned	3 & 6 month	No
Villanueuva-Vilchis (2019) (Mexico) [24]	385	Instructions about diet and oral self-care and daily toothbrushing for 1 month	Conventional dental instruction (CDI)- led by paediatric dentist	Nominated by teachers (based on their academic achievement& socialising)	3 months	Lay Advisors Model
Karimy et al. (2020) (Iran) [25]	365	Four (1 h) educational sessions weekly plus planning toothbrushing	Dental research staff-led	Nominated by peers (then interviewed to evaluate interest & suitability)	2 months	Planned Behaviour theory
Karami et al. 2019 (Iran) [26]	120	Oral Health Education (OHE), 10 min animations & practical training	Teacher-led	Selected by school	One month	
Xiang et al. 2022 (Hong Kong) [27]	1184	Six sessions: OHE Booklets & toothbrushes	Self-learning	Selected by teacher in charge	6&12 months	SCT & Health Belief Model
Aleksejeniene & Pang, 2022 (Canada) [28]	372	1 month: Lecture-based presentation followed by 4 peer-led OHE & practical sessions	Dental hygienist-led	Random selection	8 &12 months	Lay Advisors Model

### Quality assessment

Quality Appraisal for Diverse Studies (QuADS) [29] the refined version of the Quality Assessment Tool for Studies with Diverse Designs (QATSDD), was used to assess the risk of bias and overall quality of the included studies. This tool enables assessment when a range of study designs are included. The tool assesses 13 domains: theoretical underpinning to the research, statement of research aims, research setting and population, study design, sampling, rationale for choice of data collection tools, format and content of data collection tool, description of data collection procedure, recruitment data, justification of analytic method, appropriateness of analytic method in relation to research question, stakeholder involvement, strengths and limitations of research. Two reviewers (YE and AT) independently assessed the quality of the included studies following the guidance provided by authors of QuADS tool. Papers identified through an updated search (2021–2022) were reviewed by Y.E. To ensure consistency, a third of the papers from each set were cross-checked by another reviewer.

### Data synthesis

Simple descriptive analysis was conducted. Meta-analyses could not be undertaken due to the heterogeneity of intervention outcomes. Using the principles of the Synthesis Without Meta-analysis (SWiM) guideline [30], the included studies were grouped by outcome measures studied and their effect on oral health to facilitate comparisons between peer-led delivery and other delivery modes. These outcome groups included, DMFT (Decay, Missing, Filled Teeth), oral hygiene, oral health knowledge, oral health behaviour/practice, oral health attitude/ intention and quality of oral self-care/ skills.

## Results

**Fig. 1:**
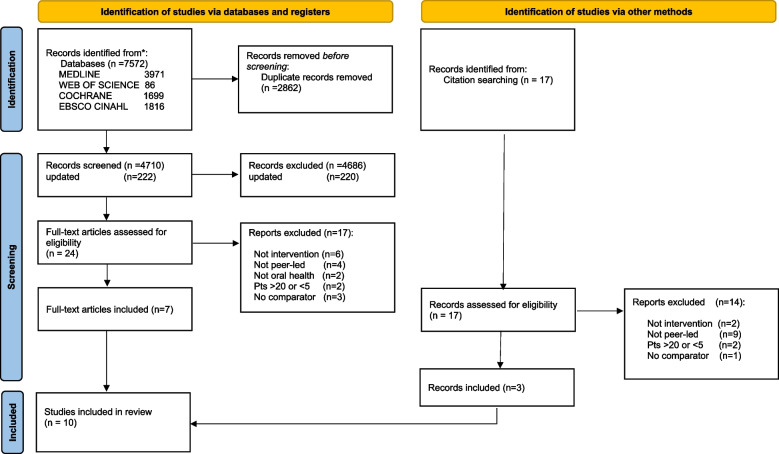
Prisma flow diagram of included studies

The systematic search of the literature on student-led school-based oral health interventions yielded 7572 records. Removal of duplicates and preliminary screening of titles and abstracts left 24 studies for full-text screening. Seven of these 24 remaining studies were included in the systematic review. Additionally, 17 studies were retrieved from citation searching of the included studies, a further three of these met the inclusion criteria. A total of ten studies were included in the systematic review. The study selection process has been clearly outlined in Fig. 1.

### Study characteristics

Ten studies comparing peer-led and professional-led oral health interventions were found. Five were randomized controlled trials, one descriptive study, two quasi-experimental studies, and two nonrandomized controlled trials. The control/comparator groups were dental professional-led groups in eight of the included studies [19–24, 26, 28], teacher-led groups in 3 studies [20, 21, 25] and self-learning groups in 3 studies [19, 20, 27] The studies took place in a variety of contexts, including Pakistan [20, 21], India [23], Indonesia [22], Iran [25, 26], Mexico [24], Hong Kong [27], Canada [28], and Finland [19].

The systematic review collectively included 5755 children with an age range of 9–15 years from a variety of schools (both private and public). The age dynamic between peer-leaders and recipients was same age in 5 studies [20, 21, 23, 26, 27] and across ages, where peer-leaders were older than recipient peers, in the rest of the studies [19, 24, 25, 28]. Although Vangipuram et al., [23] reports that the peer-leaders were chosen from the population, they include a wide age range (12–15 years old) which may imply across age peer-led education.

### Selection of peer-leaders

The student peer-leaders were selected by the schoolteachers in all the included studies except Karimy et al., [26] where the peer-leaders were nominated by their peers and then interviewed by the teachers to evaluate their interest and suitability, Aleksejuniene & Pang., [28] randomly selected peer-leaders.

### Training of peer-leaders

All studies reported peer-leaders training by researchers or professionals in charge except Laiho et al., [19] where the peer-leaders planned their own sessions with teachers' assistance. Whilst some studies did not report the duration of the training [24–26], others reported different durations varying from: short 20-min sessions [23], five 2-h sessions [20, 21], a two-day training course [27] to 4 days of training before delivering the intervention [22]. Vangipuram et al., [23] highlighted that the peer-leaders practiced three-times per week before delivering to the whole classroom. Similarly, Aleksejuniene & Pang., [28] reported that two oral health professionals assessed the peer-leaders’ oral health knowledge and oral self-care skills following the single training session they received prior to providing the peer-led session to their younger peers.

Follow up of the interventions varied from one month [22, 25, 28], 2 months [19, 26], 3 months [24], and 6 months [23, 27] to around 2 years [20, 21].

### Comparing oral health outcomes

#### Knowledge, attitude, and behaviour

##### Knowledge

Seven out of ten included studies reported change in oral health knowledge of participants, three studies did not report this measure [24, 26, 27]. However, although Xiang et al., [27] did not measure oral health knowledge per se, they measured changes in Health Belief Model (HBM) and Social Cognitive Theory (SCT) constructs (perceived susceptibility, perceived barriers, perceived benefits, perceived severity, cues to action, self-efficacy, behavioural capability, and social support) using a previously validated 42-item questionnaire. Their findings highlighted that the perceptions and other psychological constructs improved significantly in the theory-based peer-led group at 6- and 12-month follow-up except for perceived severity.

Haleem et al., [20] highlighted that all educator-led groups had significantly higher mean knowledge than the self-learning and control groups. However, comparatively they found peer-led education to be almost as effective as dentist-led but more effective than teacher led and control groups. Similarly, Vangipuram et al., [23] found that knowledge regarding the need to change toothbrushes on a regular basis increased in both dentist and peer-led groups at 6 months compared to baseline. Moreover, Haleem et al., [21] reported 26% oral health knowledge gain at 12-month follow-up of repeated and reinforced Oral Health Education (OHE) compared to their baseline knowledge. There were insignificant differences regarding these findings between peer-led, dentist-led, and teacher-led groups. Debby et al., [22] report a meaningful knowledge gain following peer supported education in the experimental group. However, they also found a similarly significant knowledge gain within their control group who received education via traditional lecturing methods. No method was reported as superior with both impacting oral health knowledge significantly. Laiho et al., [19] found all three interventional teaching methods (peer-led, dentist-led, and self-led) were moderately successful in increasing knowledge of oral diseases, however, significant gaps in knowledge were reported pre and post intervention in the opinion and knowledge of prevention segments of the questionnaire. The authors reported all three intervention methods were ineffective in increasing the knowledge of measures to be taken in oral self-care, they concluded peer OHE was the most effective method of delivery yet report significant heterogeneity of knowledge gains across a variety of topics. Karami et al., [25] reported significant improvement in knowledge of both peer-led and teacher led groups, yet the knowledge gain was more in the peer-led group. Aleksejuniene & Pang., [28] reported significant increase in knowledge of cariogenic diets from baseline in the peer-led group compared to insignificant change in the control group. The authors highlighted the participants in both groups had similar baseline knowledge with less than 25% knowing the main cause of caries.

##### Attitude

Five of the included studies reported changes in oral health attitudes and/or intention [21–23, 25, 26]. Laiho et al., [19] investigated the attitudes of participants towards the oral health sessions but not direct behaviours which would impact health outcomes.

Haleem et al., [21] found that neither single oral health education session nor repetitive reinforced sessions changed children’s attitude towards their oral hygiene practice regardless of mode of delivery. Nonetheless, they did highlight that participant from all groups showed positive attitudes towards oral hygiene maintenance at baseline. Vangipuram et al., [23] reported improvements in attitudes toward oral hygiene, in both peer-led and dentist-led groups. Karimy et al., [26] found a significant improvement following intervention delivery between the mean and SD in numerous domains relating to OH attitude. They reported a statistically significant improvement in attitude, subjective norms, and intention in the intervention group at follow-up compared to baseline, providing evidence for the use of theory of planned behaviour in changing oral health related attitudes. They suggest their results indicate that providing structure for participants to plan their time, place, and method for brushing could impact consistent brushing behaviour. Debby et al., [22] found a positive change in attitude after the peer-support education intervention. Similarly, Karami et al., [25] found a significant increase in attitude scores before and after the intervention in both teacher-led and peer-led groups.

##### Behaviour/hygiene practice

Six studies measured oral health behaviours and practices [20, 21, 23, 25–27], they collected data on methods, frequency and duration of oral health behaviours/practices including dietary behaviours and dental attendance. The remaining four studies did not report behaviours and practices as their outcome measures [19, 22, 24, 28].

Haleem et al., [20] highlighted that all intervention groups had significantly higher mean behaviour than the control and self-learning group, yet peer-led group showed significantly better oral health behaviours than the teacher-led. Likewise, Vangipuram et al., [23] reported the same compared to their dentist-led counterparts. Findings from Haleem et al., [21] demonstrated statistical superiority of reinforced OHE in improving oral health behaviours with all modes of delivery. Karimy et al., [26] found the intervention impacted student toothbrushing behaviour, with an increase in the rate of twice day toothbrushing and flossing (both statistically significant), they found that action planning and coping were important variables to impact these behaviours and report a doubling of flossing behaviour in the experimental group. They found that a peer-led approach was more influential than the adult-led approaches in enhancing toothbrushing behaviour. Similarly, Karami et al., [25] reported a significant increase in toothbrushing, flossing, use of mouthwash and regular visits to the dentist in both teacher-led and peer-led groups post-intervention. The authors do conclude that overall, the peer-led approach was more successful than the teacher led approach in improving oral health knowledge, attitudes, and behaviours, when adjusting the parent’s job variable. Whereas Xiang et al., [27] found that peer-led group showed a statistically significant improvement in the frequency of toothbrushing and flossing. Although the improvement was apparent in the short-term, authors reported that it was sustained short-term improvement after 12 months.

#### Oral health status

##### Oral hygiene

Four studies reported on oral hygiene status, utilising plaque indices and bleeding on probing, [20, 21, 23, 27]. Two of these studies also reported calculus [20, 21]. Two additional studies, however, utilised plaque levels to measure the quality of oral self-care [24, 28], yet the remaining included studies did not measure oral hygiene status of participants.

Haleem et al., [20] noted that dentist-led, teacher-led and peer-led OHE were found to be equally effective in improving oral hygiene status. Similarly, Vangipuram et al., [23] showed a significant reduction in mean plaque and gingival scores following both peer- and dentist-led OHE interventions. However, Haleem et al., [21] reported no change in oral hygiene status in all groups following a single oral health education session, irrespective of who led the intervention. They suggested that repetition/reinforcement of the OHE was more important than who delivered the OHE. Xiang et al., [27] measured plaque using a Visual Plaque Index, reported larger reduction in plaque scores in the peer-led group at 12-month follow-up compared to the control group.

##### DMFT (Decay, Missing, Filled Teeth Index)

From the ten included studies, only two studies [21, 27] reported DMFT as an outcome measure. Likewise, Haleem et al., [21] found negligible difference in DMFT scores in all groups following both single session OHE and repeated and reinforced OHE. However, it was acknowledged that the study participants showed low DMFT levels at the start of the study which reflects the negligible change. Nevertheless, Xiang et al., [27] reported a statistically significant decline in DMFT scores (p < 0.001) in the peer-led intervention group at the 12-month follow-up.

#### Quality of oral self care

Two included studies [24, 28] identified quality of oral self-care practice and skills as their main outcome measures. These were measured using disclosing solution and consequently assessing plaque levels. Findings of Villaneuva-Vilchis et al., [24] reported significant improvement in oral self-care practices and skills (i.e., decrease in plaque levels) in peer-led intervention group compared to those receiving conventional oral health education. This finding suggests that peer-led OHE improved plaque status as a consequence of improved toothbrushing when compared to conventional OHE. Although Aleksejuniene & Pang., [28] reported short-term improvements in oral self-care, they highlighted considerable decrease in the percentage of biofilm post-intervention in both study groups, compared to baseline. Similarly, there was a significant decrease (p > 0.001) in the intervention group versus the control.

#### Oral Health Related Quality of Life (OHRQoL)

Only one of the included studies reported changes in OHRQoL, they used a shortened 16-item Child Perceptions Questionnaire (CPQ11-14). They reported a significant improvement in OHRQoL which was sustained at the 12-month follow-up [27]. Table 3 provides a comparison of outcomes in the included studies.

**Table 3:** Comparison of outcome measures reported in included studies

	**Clinical Measures**		**Knowledge**	**Attitudes & behaviours**		**Skills**	**OHRQoL**
Main Outcomes:	**DMFT**	**Oral Hygiene** **(PI, BOP, Cal)**	**OH Knowledge**	**OH Behaviour/ Practice**	**OH Attitude/ Intention**	**Quality of Oral Self Care/** **Skills**	
**Haleem et al., 2012 **[20]	N	Y	Y	Y	N	N	N
**Vangipuram et al., 2016** [23]	N	Y	Y	Y	Y	N	N
**Villaneuva-Vilchis, 2019** [24]	N	N	N	N	N	Y *(p* < *0.001)*	N
**Haleem et al., 2015 **[21]	Y	Y	Y	Y	Y	N	N
**Karimy et al., 2020** [26]	N	N	N	Y	Y	N	N
**Debby et al., 2016** [22]	N	N	Y	N	Y	N	N
**Laiho et al., 1993** [19]	N	N	Y	N	N^a^	N	N
**Xiang et al., 2022** [27]	Y *(p* < *0.001)*	Y	N	Y	N	N	Y
**Aleksejuniene & Pang, 2022** [28]	N	N	Y	Y	N	Y	N
**Karami et al., 2019** [25]	N	N	Y	Y	Y	N	N

^a^ (attitude to education not OH)

### Quality of evidence

A quality assessment of included studies was undertaken using the QuADS assessment tool [29]. As described by the authors, the use of numerical criteria to report the quality of included studies is considered arbitrary. Users of the tool are advised to consider the quality assessment process narratively and within the context of their own research.

The majority of the studies scored high according to QuADs criteria. Generally, all studies provided clear descriptions of aims and objectives with appropriately selected methodologies. All but one study demonstrated appropriate sampling strategies including the use of powered sample size calculations [22]. Whilst Debby et al., [22] did not utilise sample size calculations, the sampling strategy was well defined; 70 participants were included in the intervention, a number much smaller than all the other studies.

Additionally, six out of the ten articles included in this review utilised behavioural and psychological theory as the theoretical underpinning of their intervention. The theories in this review included the SCT [20], Social Learning Theory [21], Planned Behaviour Theory [26], Lay health advisors’ model [24, 28]. Xiang et al. [27] was a multi-theory-based intervention guided by the HBM and SCT.

There was a clear lack of stakeholder involvement in most studies, however, four studies mentioned stakeholder consultations when the planning the interventions [20, 22, 27, 28]. No co-production i.e., development of the study with young people or key stakeholder was reported in any of the studies.

The quality of the discussion of the strengths and limitations varied across all studies. Most studies provided a limited summary of strengths and limitations, however, their analysis lacked depth of explanation. Five studies [20, 21, 24, 27, 28] provided more comprehensive and complete analysis including discussion of study design, methods and analysis.

The complete quality assessment table can be found in Additional file 2.

## Discussion

### Summary of key findings

This systematic review examined the change in oral-health outcome measures following school-based student peer-led oral health interventions when compared to other modes of delivery. Although the search strategy identified numerous interventional studies, only 10 studies were included as they compared peer-led interventions to other modes of delivery rather than no intervention. All the included studies demonstrated that peer-led school-based oral health interventions reported the same, and in some instances, a greater improvement in oral health outcomes compared to professional-led interventions. These confirmed findings of a critical review comparing peer-led to adult led delivery [12].

The peer-led approach has been extensively researched in evidence-based literature across multiple health disciplines. Recent systematic reviews examining this approach in school settings were found in different topics such as mental health [14, 31], nutrition [15], physical activity (PA) [13], and tobacco, alcohol, and drug use [11]. Findings of these reviews indicated that peer-led interventions can improve health outcomes yet highlighted sustainability and scalability issues [15].

Health interventions have the scope to widen health inequalities. This can be seen in the ‘inverse prevention’ or ‘inverse care’ laws [32] whereby those most in need of the services are the least likely to receive them. The recent Commissioning Better Oral Health for Children and Young People recommends the investigation of community/peer-led programmes which facilitate improvements in oral health [33]. Other studies have encouraged community involvement whilst tackling cultural barriers, ensuring access to those often ‘hard to reach’ and engaging intervention communities to build sustainable and scalable peer-led programmes [16, 34].

This review provides further insight into whether peer-led interventions can impact on oral health of school children and remove any of the traditionally identified barriers to implementing school-based oral health intervention. These challenges include lack of resources, lack of continuity, lack of ownership, and increasing cost of delivery. Some of the included studies highlighted the superiority of peer-delivery as a cost effective and scalable mode of delivery [20, 21, 28]. This has been reiterated in recent reviews of peer-delivered interventions promoting health-enhancing physical activity [13, 15, 35]. Nevertheless, further research into the barriers and facilitators from the students and teacher’s perspectives has been recommended to allow feasible maintenance and areas for improvement of intervention design and delivery [13, 15].

The school environment provides numerous opportunities to improve the health behaviours in children [36, 37]. School-based interventions, especially those targeting deprived areas, are suggested to improve oral health equity [38]. Oral health related behaviours in school-based interventions have traditionally been confined to educational sessions delivered by teachers and/or professionals. Previous reviews of school-based interventions promoting oral health behaviours of children highlighted the importance of peer-led delivery and its potential for success [39, 40].

Peer-leaders require adult support and training for successful engagement with their peers and intervention delivery. The review identified various lengths and method of peer-leader training, yet the many studies lacked comprehensiveness and clarity in describing the training process. Henceforth, emphasis should be made on clear planning and description of peer-leader training and delivery in future work. According to the findings of this review, both across-age and same-age dynamics of peer-led delivery have been found effective and acceptable in young adolescents. Nonetheless, evidence of programmes targeting older adolescents was sparse. A finding similar to a recent systematic review of PA interventions [13]. Qualitative research with the peer-leaders suggested a wider age gap between peer-leaders and their recipient peers would be preferable [28]. However, they highlight that working with younger peers can be frustrating yet empowering. It has been pointed out that student opinion is important in the selection process of peer-leaders rather than teacher selection to empower the students to take part and feel involved [13, 28]. The above-mentioned factors are of vital importance for the success of the intervention and to guide future work regarding the mutual benefit of this mode of delivery.

Another important point to be considered when delivering school-based interventions is the theoretical foundation that informs intervention delivery. Five psychological theoretical frameworks were the basis of six studies in this review. The SCT was the most frequently used and considered an appropriate approach for peer-led prevention [41]. The SCT has also been suggested as an ideal approach for promoting healthy behaviours in adolescents [42, 43]. However, there is lack of strong evidence supporting this method of education because of the difficulty in controlling the confounding factors. Xiang et al., [44] suggested that future research should aim to identify appropriate Behaviour Change Techniques (BCTs) with detailed description of the techniques used, long-term follow-up and provision of reinforcement sessions to optimise oral health behaviour change in peer-led interventions. Nevertheless, regardless of the approach taken, oral health promotion using peer-leadership, within an education setting capturing a full social ecological model [45] can show promising results with adolescents.

It has been concluded in previous literature that oral health education alone has no discernible effect on dental caries particularly on the long term [39, 40, 46]. Xiang et al., [27] reported statistically significant decrease in DMFT and plaque one-year post-intervention. However, Haleem et al., [21] reported a negligible difference in DMFT at 2-year follow-up. This highlights the significance of sound theoretical basis when planning and implementing oral health programmes as Xiang et al., [27] was a multi-theory-based peer-led intervention.

### Strengths and limitations

This is the first systematic review to evaluate and compare the oral health outcomes of peer-led school-based interventions versus other modes of delivery. A strength of this review was that calibration, pilot data extraction and quality assessment were undertaken with high intra- and inter-rater agreements scores achieved. Continuous involvement of experts in the field at each stage was also considered a strength of this review.

Whilst there may be a possibility that relevant studies were overlooked, this possibility was reduced by two reviewers replicating the search independently and considering the search strategy was informed by subject experts and librarians. However, due to the broad range of study designs and study outcomes (heterogeneity), a quantitative synthesis (meta-analysis) was not possible. Additionally, it should be acknowledged that the use of a non-study design specific quality assessment tool such as the QuADS tool [29] may be of disadvantage particularly if results were reported narratively as recommended by authors of this tool. This may cause a possibility to miss out on the variations between study designs or influence the ability to distinguish between the quality of included studies in some respects.

In terms of generalisability and applicability of the results of this review, it could still be of relevance for other school-based student-led peer delivered health promotion approaches. The review included a wide range of developed and developing countries from different parts of the world. Further, as mentioned above, many other peer-led interventional studies were found yet excluded as they lacked a comparator. These studies were conducted in many counties such as, to name a few, the UK [47], Lithuania [48], and Germany [49]. The absence of UK-based studies may reflect a lack of this approach to oral health promotion in schools emphasising only the educational aspect rather than involving the students themselves in order to achieve behaviour change [12].

## Conclusion

All the included studies demonstrated that oral health outcomes of peer-led school-based oral health interventions reported either no change or improvement in oral health outcomes.

Future research should consider the complex nature of school-based interventions and the requirement for large sample sizes to achieve scalability, address the generalisation of results, and aid implementation. Findings of this review also recommend future focus on the bi-directional impact of peer-led interventions and added value to peer-leaders as intervention providers.

### Supplementary Information

**Additional file 1. **MEDLINE via Ovid Search Strategy.

**Additional file 2.** Quality assessment of included studies.

**Additional file 3.**

## Data Availability

All data generated or analysed during this study are included in this published article [and its supplementary information files.

## References

[1] Bernabe E, Marcenes W, Hernandez C, Bailey J, Abreu L, Alipour V (2020). Global, regional, and national levels and trends in burden of oral conditions from 1990 to 2017: a systematic analysis for the global burden of disease 2017 study. J Dent Res.

[2] Peres M, Macpherson L, Weyant R, Daly B, Venturelli R, Mathur M, Lislt S, Celeste RK, Guarnizo-Herreño Kearns C, Benzian H, Allison P, Watt RG (2019). Oral diseases: a global public health challenge. Lancet.

[3] Godson J, Csikar J, White S (2018). Oral health of children in England: a call to action!. Arch Dis Child.

[4] Office for Health Improvement and Disparities (OHID) (2022). Child oral health: applying All Our Health.

[5] Pitts NB, Chadwick B, Anderson T. Children's Dental Health Survey 2013. Report 2: Dental disease and damage in children England, Wales and Northern Island England: Health & Social Care Information Centre. 2015

[6] Herlitz L, MacIntyre H, Osborn T, Bonell C (2020). The sustainability of public health interventions in schools: a systematic review. Implement Sci.

[7] Tjomsland HE, Larsen TMB, Viig NG, Wold B. A fourteen year follow-up study of health promoting schools in Norway: principals’ perceptions of conditions influencing sustainability. Open Educ J. 2009;2(1):54–64.

[8] Cowie H, Hutson N, Oztug O, Myers C (2008). The impact of peer support schemes on pupils' perceptions of bullying, aggression and safety at school. Emot Behav Diffic.

[9] Simoni JM, Franks JC, Lehavot K, Yard SS (2011). Peer interventions to promote health: conceptual considerations. Am J Orthopsychiatry.

[10] Lindsey B (1997). Peer education: a viewpoint and critique. J Am Coll Health.

[11] MacArthur GJ, Harrison S, Caldwell DM, Hickman M, Campbell R (2016). Peer-led interventions to prevent tobacco, alcohol and/or drug use among young people aged 11–21 years: a systematic review and meta-analysis. Addiction.

[12] Mellanby AR, Rees JB, Tripp JH (2000). Peer-led and adult-led school health education: a critical review of available comparative research. Health Educ Res.

[13] McHale F, Ng K, Taylor S, Bengoechea E, Norton C, O’Shea D (2022). A systematic literature review of peer-led strategies for promoting physical activity levels of adolescents. Health Educ Behav.

[14] King T, Fazel M (2019). Examining the mental health outcomes of peer-led school-based interventions on young people aged between 4 and 18 years old: a systematic review protocol. Syst Rev.

[15] Yip C, Gates M, Gates A, Hanning R (2016). Peer-led nutrition education programs for school-aged youth: a systematic review of the literature. Health Educ Res.

[16] Dodd S, Widnall E, Russell AE, Curtin EL, Simmonds R, Limmer M (2022). School-based peer education interventions to improve health: a global systematic review of effectiveness. BMC Public Health.

[17] Elsadek Y, Edwebi, S., Turner, A., Vinall-Collier, K., Pavitt, S & Csikar, J. A systematic review of school-based student-led oral health interventions to promote oral health of primary schoolchildren. PROSPERO 2021 CRD42021283542. Available from: https://www.crd.york.ac.uk/prospero/display_record.php?ID=CRD42021283542 . 10.1186/s12903-023-03482-1 PMC10566183 37817155

[18] Page M, McKenzie J, Bossuyt P, Boutron I, Hoffmann T, Mulrow C (2021). The PRISMA 2020 statement: an updated guideline for reporting systematic reviews. Int J Surg.

[19] Laiho M, Honkala E, Nyyssönen V, Milen A (1993). Three methods of oral health education in secondary schools. Eur J Oral Sci.

[20] Haleem A, Siddiqui MI, Khan AA (2012). School-based strategies for oral health education of adolescents-a cluster randomized controlled trial. BMC Oral Health.

[21] Haleem A, Khan MK, Sufia S, Chaudhry S, Siddiqui MI, Khan AA (2015). The role of repetition and reinforcement in school-based oral health education-a cluster randomized controlled trial. BMC Public Health.

[22] Debby Syahru R, Taufan B, Muhammad L (2016). The effect of peer support education on dental caries prevention behavior in school age children at age 10–11 years old. Dent J (Majalah Kedokteran Gigi).

[23] Vangipuram S, Jha A, Raju R, Bashyam M (2016). Effectiveness of peer group and conventional method (dentist) of oral health education programme among 12–15 year old school children-a randomized controlled trial. J Clin Diagn Res.

[24] Villanueva-Vilchis MdC, Aleksejūnienė J, López-Núñez B (2019). A peer-led dental education program for modifying oral self-care in Mexican children. Salud Pública Méx.

[25] Karami A, Heidarnia A, Zarei F (2019). Comparison of peer led and teacher led oral health educational program among students. Braz J Oral Sci.

[26] Karimy M, Higgs P, Abadi SS, Armoon B, Araban M, Rouhani MR (2020). Oral health behavior among school children aged 11–13 years in Saveh, Iran: an evaluation of a theory-driven intervention. BMC Pediatr.

[27] Xiang B, McGrath CP, Wong HM (2022). The Efficacy of a multi-theory-based peer-led intervention on oral health among Hong Kong Adolescents: A Cluster-Randomized Controlled Trial. J Adolesc Health.

[28] Aleksejuniene J, Pang RHI (2022). Peer-led oral health education model for elementary school-aged children in British Columbia, Canada. Can J Dent Hygiene.

[29] Harrison R, Jones B, Gardner P, Lawton R (2021). Quality assessment with diverse studies (QuADS): an appraisal tool for methodological and reporting quality in systematic reviews of mixed-or multi-method studies. BMC Health Serv Res.

[30] Campbell M, McKenzie JE, Sowden A, Katikireddi SV, Brennan SE, Ellis S (2020). Synthesis without meta-analysis (SWiM) in systematic reviews: reporting guideline. BMJ.

[31] Mackenzie K, Williams C (2018). Universal, school-based interventions to promote mental and emotional well-being: What is being done in the UK and does it work? A systematic review. BMJ Open.

[32] Harris R (2016). Do'poor areas' get the services they deserve? The role of dental services in structural inequalities in oral health. Community Dent Health.

[33] Yusuf H, Wright K, Robertson C (2015). Evaluation of a pilot oral health promotion programme'Keep Smiling': perspectives from GDPs, health champions and school staff. Br Dent J.

[34] Chowdhary P, Mekuria F, Tewahido D, Gulema H, Derni R, Edmeades J (2022). Building sustainable and scalable peer-based programming: promising approaches from TESFA in Ethiopia. Reprod Health.

[35] Hulteen RM, Waldhauser KJ, Beauchamp MR (2019). Promoting health-enhancing physical activity: a state-of-the-art review of peer-delivered interventions. Curr Obes Rep.

[36] Kwan SY, Petersen PE, Pine CM, Borutta A (2005). Health-promoting schools: an opportunity for oral health promotion. Bull World Health Organ.

[37] Langford R, Bonell CP, Jones HE, Pouliou T, Murphy SM, Waters E, et al. The WHO Health Promoting School framework for improving the health and well‐being of students and their academic achievement. Cochrane Database Syst Rev. 2014(4):CD008958. 10.1002/14651858.CD008958.pub2. 10.1002/14651858.CD008958.pub2 PMC11214127 24737131

[38] Gargano L, Mason MK, Northridge ME (2019). Advancing oral health equity through school-based oral health programs: An ecological model and review. Front Public Health.

[39] Cooper AM, O'Malley LA, Elison SN, Armstrong R, Burnside G, Adair P, et al. Primary school‐based behavioural interventions for preventing caries. Cochrane Database Syst Rev. 2013(5):CD009378. 10.1002/14651858.CD009378.pub2. 10.1002/14651858.CD009378.pub2 PMC11873670 23728691

[40] Joury E, Shakir A, Barngkgei I, Godson J (2021). Effectiveness of school-based behavioural interventions to improve children’s oral health by reducing sugar intake and promoting oral hygiene: A rapid review of randomised controlled trials. Community Dent Health.

[41] Bandura A (1986). Social foundations of thought and action.

[42] Chilton JM, Haas BK, Gosselin KP (2014). The effect of a wellness program on adolescent females. West J Nurs Res.

[43] Dewar DL, Plotnikoff RC, Morgan PJ, Okely AD, Costigan SA, Lubans DR (2013). Testing social-cognitive theory to explain physical activity change in adolescent girls from low-income communities. Res Q Exerc Sport.

[44] Xiang B, Wong HM, Perfecto AP, McGrath CP (2020). The effectiveness of behavioral interventions to improve oral health in adolescents at different periods of follow-up: a systematic review and meta-analysis. Patient Educ Couns.

[45] Bronfenbrenner U (1986). Ecology of the family as a context for human development: Research perspectives. Dev Psychol.

[46] de Silva AM, Hegde S, Nwagbara BA, Calache H, Gussy MG, Nasser M (2016). Community-based population-level interventions for promoting child oral health. Cochrane Database Syst Rev.

[47] Yeo KY, Hashimoto K, Archer T, Kenny K, Pavitt S, Zoltie T (2020). Evaluation on the effectiveness of a peer led video on oral hygiene education in young children. J Vis Commun Med.

[48] Aleksejūnienė J, Brukienė V, Džiaugyte L, Pečiulienė V, Bendinskaitė R (2016). A theory-guided school-based intervention in order to improve adolescents' oral self-care: a cluster randomized trial. Int J Pediatr Dent.

[49] Reinhardt CH (2009). Peer teaching pilot programme for caries prevention in underprivileged and migrant populations. Int J Pediatr Dent.

