# Supplementary material for: A systematic review of school-based student peer-led oral health interventions to promote the oral health of school children
Source: BMC Oral Health. 2023 Oct 10;23:742. doi: 10.1186/s12903-023-03482-1 (PMC10566183; doi:10.1186/s12903-023-03482-1)
Supplement: Supplementary file 1 — Additional file 1. MEDLINE via Ovid Search Strategy. [file 12903_2023_3482_MOESM1_ESM.docx]

**Additional file 1.** MEDLINE via Ovid Search Strategy

**ORAL HEALTH**

1. (caries or cario* or decay*).mp.

2. “dental plaque”.mp

3. toothache.mp

4. “Tooth decay”.mp

5.exp Dental Health Surveys/

6.("DMF Index" or "dental plaque index" or "oral hygiene index").mp.

7. (Periodont* or gingiv* or gum*).mp

8.exp Oral Health/ or “oral health”. mp

9.exp Oral Hygiene/

10. (Toothbrush* or "tooth brush*" or tooth-brush* or toothpaste* or tooth-paste* or "tooth paste" or dentifric*).mp.

11. (Mouthwash* or mouthrinse* or "mouth wash*" or "mouth rinse*" or "mouth-wash*" or "mouth-rinse*").mp.

12. ("sugar intake" or sweet* or candy or candies or gum*).mp.

13. (snack* or diet* or food* or drink* or beverage*).mp.

14. “dental health” .mp.

15. or/1-14

**SCHOOL**

16. Schools/ or school*.mp

17. College*.mp

18. Exp “Academies and institutes”/

19. exp “School health services”/

20.or/16-19

**STUDENT-LED**

21. Student adj4 (led or run or based or support or facilitated or directed or managed or mediat* or tutor* or influence).mp

22. Pupil adj4 (led or run or based or support or facilitated or directed or managed or mediat* or tutor* or influence).mp

23. Peer adj4 (led or run or based or support or facilitated or directed or managed or mediat* or tutor* or influence).mp

24. Train the trainer.mp

25. “Cross-age”.mp

26. mentors/ or Mentor*.mp

27. Or/21-26

**INTERVENTION**

28. Health education, dental/ or Health education /

29. Health promotion/

30. Health behavior/

31. [Health Knowledge, Attitudes, Practice/](https://ovidsp.dc1.ovid.com/ovid-b/ovidweb.cgi?&Controlled+Vocabulary=Mapping%7c1&Return=mapping&S=BMILFPIMAKACNMOBKPOJLEDKNALEAA00)

32. (instruct* or advice or advis* or educat* or teach* or train* or promot* or prevent* or interven* or supervise* or scheme or program*).mp.

33. Or/28-32

**POPULATION**

34. Child/ or Child*.mp.

35. Adolescent/ or Adolescen*.mp.

36. Teen*.mp

37. Or/34-36

38. 15 and 20 and 27 and 33 and 37
