# Supplementary material for: A systematic review of school-based student peer-led oral health interventions to promote the oral health of school children
Source: BMC Oral Health. 2023 Oct 10;23:742. doi: 10.1186/s12903-023-03482-1 (PMC10566183; doi:10.1186/s12903-023-03482-1)
Supplement: Supplementary file 2 — Additional file 2. Quality assessment of included studies. [file 12903_2023_3482_MOESM2_ESM.docx]

**Additional file 2.** Quality assessment of included studies

| Author | 1. Theoretical underpinning | 2. Statement of aim/s | 3. research setting and target population | 4. Appropriate study design | 5.Appropriate sampling to address the research aim/s | 6. Rationale for choice of data collection tool/s | 7. appropriate data collection tools | 8. Description of data collection procedure | 9. Recruitment data provided | 10. Justification for analytic method | 11. Appropriate method of analysis | 12. Stakeholders involvement | 13. Strengths and limitations |
| --- | --- | --- | --- | --- | --- | --- | --- | --- | --- | --- | --- | --- | --- |
| Haleem et al 2012 | 2 | 3 | 3 | 3 | 3 | 3 | 3 | 3 | 3 | 3 | 3 | 2 | 3 |
| Rescreen | 2 | 3 | 3 | 3 | 3 | 3 | 3 | 3 | 3 | 3 | 3 | 2 | 3 |
| Vangipuram et al 2016 | 0 | 3 | 3 | 3 | 3 | 3 | 3 | 3 | 3 | 3 | 2 | 1 | 2 |
| Rescreen | 0 | 3 | 3 | 3 | 3 | 3 | 3 | 3 | 3 | 3 | 2 | 1 | 1 |
| Villaneuva-Vilchis 2019 | 3 | 3 | 3 | 3 | 3 | 3 | 3 | 3 | 3 | 3 | 3 | 0 | 3 |
| Haleem et al 2015 | 2 | 3 | 3 | 3 | 3 | 3 | 3 | 3 | 3 | 3 | 3 | 2 | 3 |
| Karimy et al 2020 | 3 | 2 | 2 | 3 | 3 | 3 | 3 | 2 | 2 | 3 | 3 | 0 | 2 |
| Debby et al 2016 | 1 | 2 | 3 | 2 | 2 | 2 | 2 | 2 | 2 | 2 | 3 | 2 | 1 |
| Laiho et al 1993 | 1 | 2 | 3 | 3 | 3 | 2 | 2 | 2 | 3 | 2 | 3 | 1 | 1 |
| Xiang et al 2022 | 3 | 3 | 3 | 3 | 3 | 2 | 3 | 3 | 3 | 2 | 2 | 3 | 3 |
| Aleksejuniene & Pang 2022 | 3 | 3 | 3 | 2 | 3 | 3 | 3 | 3 | 3 | 3 | 3 | 3 | 3 |
| Karami et al 2019 | 1 | 3 | 3 | 1 | 3 | 3 | 2 | 3 | 3 | 1 | 2 | 0 | 1 |

| Key*:  0 – Not mentioned at all  1 – General  2 – Identified  3- Explicit and detailed |
| --- |

*See additional file 3 for QuADS 2021 detailed scoring criteria
